# Diagnosis and staging of superficial esophageal precursor based on pre-endoscopic resection system comparable to endoscopic resection

**DOI:** 10.1186/1471-2407-14-774

**Published:** 2014-10-21

**Authors:** Xiumin Qin, Shun He, Yueming Zhang, Liyan Xue, Ning Lu, Guiqi Wang

**Affiliations:** Department of Endoscopy, Cancer Institute & Hospital, Chinese Academy of Medical Sciences, Peking Union Medical College, Beijing, China; Department of Pathology, Cancer Institute & Hospital, Chinese Academy of Medical Sciences, Peking Union Medical College, Beijing, China

**Keywords:** Endoscopic resection (ER), Endoscopic treatment, Superficial esophageal neoplasm, Histological diagnosis

## Abstract

**Background:**

Endoscopic treatments for early esophageal squamous cell carcinoma and the esophageal neoplasm are two types: endoscopic resection (ER) and ablation. Resection enables evaluation of the lesion in the ER specimens, while ablation cannot. We sought to establish a pre-ER evaluated system with a diagnostic and staging accuracy similar to ER for the development of ablation therapy.

**Methods:**

In our study, we collected data pertaining to early esophageal cancer and esophageal neoplasm treated with ER, analyzed the pre- and post-ER data of the lesions and evaluated the diagnostic accuracy of pre-ER system compared with the gold standard.

**Results:**

The diagnostic accuracy rate was 91% based on the pre-ER system compared with the gold standard, and 93% based on the ER diagnosis. The AUC of the pre-ER system was 0.964, while the ER examination was 0.971.

**Conclusion:**

These results suggest that the accuracy of pre-ER system was comparable to ER. The pre-ER system enables prediction of histological diagnosis and stage of the lesions, and the choice of treatment for superficial esophageal neoplasm.

## Background

Endoscopic resection (ER), indicated for the treatment of superficial (early) gastroesophageal precursor, is also a good diagnostic approach. According to the 2010 National Comprehensive Cancer Network (NCCN) Clinical Practice Guidelines in Oncology-Esophageal Cancer [1], esophagectomy, endoscopic resection (ER) or ablation is indicated for Tis or T1a tumors. ER specimens are used to accurately determine the histopathological diagnosis as well as the depth of tumor invasion in superficial lesions before esophagectomy. Indications for ER treatment for esophageal cancer include well and/or moderately differentiated squamous cell carcinoma (SCC) confined to the lamina propria without evidence of venous or lymphatic involvement [2, 3]. The updated Paris classification suggested that the cut-off limit of esophageal superficial lesions for endoscopic treatment was 200 μm invasive depth into the submucosa, which was also supported by our previous study [4].

The endoscopy treatment for the early esophageal cancer and precursor, may be categorized into endoscopic resection(ER) with the samples and ablation therapies without the samples. Determination of the depth of the lesions without ER or esophagectomy should be further studied. Although Endoscopic Ultrasonography (EUS) allows evaluation of the invasive depth of the lesions and the biopsy provides pathological diagnosis, appropriate treatment of early esophageal cancer and precursor should also based on endoscopic appearance and tumor grade. The pre-ER diagnosis is based on a combination of endoscopy, macroscopic type (Paris classification), EUS and biopsy. Ablation therapies such as radiofrequency ablation (RFA) and photodynamic treatment (PDT) without ER specimens after treatment constitute a meaningful approach.

In this study, we compared the diagnostic concordance of the pre-ER system and ER histological diagnosis with the gold standard in superficial esophageal precursor and evaluated the accuracies of the two approaches. In practice, ER histological diagnoses are not identical with true diagnosis. Therefore, the gold standard adopted included the worse diagnosis between biopsies and post-ER histological diagnoses.

## Methods

### Patients

We retrospectively analyzed data of the early esophageal cancer and precursor, which were treated with endoscopic resection (ER) at the Department of Endoscopy, Cancer Institute and Hospital, Chinese Academy of Medical Science from January 2007 to March 2011. The indications for ER in our hospital include: (1) Endoscopic and histological diagnosis of high-grade intraepithelial neoplasia (HGIN, severe dysplasia) or early esophageal cancer (ESCC); (2) low-grade intra-epithelial neoplasia (LGIN, mild dysplasia) or middle-grade intraepithelial neoplasia (MGIN, moderate dysplasia), while the diagnosis based on the endoscopic examination was worse, as HGIN or ESCC; (3) “superficial” (type 0-II) endoscopic appearance: “elevated” (type 0-IIa), “flat” (type 0-IIb) or “depressed”(type 0-IIc). The contraindications for ER included: (1) Lesions with positive lymph node metastasis (N) or distant metastasis (M); (2) EUS showing depth of invasion into the lower two-thirds of the submucosa; (3) “protruding” (type 0-I) or “excavated” (type 0-III) endoscopic appearance, according to the Paris classification; (4) esophageal varices at the range of the lesion; and (5) uncontrolled coagulopathy with international normalized ratio (INR) >2 or platelet count <75,000 per μL.

### Procedures

The lesions diagnosed as esophageal precursor or early cancer were stained with Lugol’s solution (if not allergic) according to the endoscopic appearance. In our study, no patients with allergy were seen, and all the lesions were stained by the Lugol’s solution. The number of the forceps biopsy was up to the length of the lesion, for instance, lesion > 1 cm corresponded with the number of the biopsy ≥ 2; the length > 2 cm, corresponded with number ≥ 3, and the length > 3 cm, the number ≥ 4. The endoscopic diagnosis was based on Lugol’s staining according to the grade of the lesions [5]. If the diagnosis based on the endoscopic images was HGIN or ESCC (that is grade I), while the histopathological diagnosis based on the biopsy was LGIN or MGIN, ER was performed after consultation with patients. The macroscopic classification was based on Paris endoscopic classification [2, 3], classified as 0-IIa, 0-IIb, 0-IIc. We used the Paris classification to distinguish the lesions as invasive or non-invasive, to decide the appropriate therapeutic intervention. Endoscopic ultrasonography (EUS) and computed tomography (CT) were performed before ER to confirm the depth and absence of metastasis. Only lesions without metastasis were appropriate for ER. If the lesions were located in the mucosa or the upper one- third of the submucosa (sm1), the lesion was treated with ER. If the lesions invaded the lower two-third of the submucosa (sm2), surgery was indicated according to the EUS diagnosis.

The ER includes the endoscopic mucosa resection (EMR) with suction cap- and/or saline solution-assisted snare resection techniques according to Haruhiro Inouen [6–8] and multiband mucosectomy (MBM) [9].

### Management of biopsy and ER specimens

Biopsy specimens were obtained from all unstained lesions (USLs) using standard biopsy forceps. Biopsy specimens were removed using a toothpick or other non-traumatic technique and spread out flat on a gloved finger. Each specimen was separately attached to filter paper and fixed immediately. The filter paper was later removed and the tissue was dehydrated, and embedded perpendicularly in paraffin.

Resection specimens were pinned onto the cork with the luminal side facing up. Piecemeal resection specimens were reconstructed after staining with Lugol’s solution. Specimens were subsequently fixed in 10% buffered formalin, cut into 2 mm sections, dehydrated and embedded perpendicularly in paraffin.

Slices were cut, mounted on glass slides and stained with hematoxylin and eosin. All slides of the biopsy and the ER specimens were reviewed independently by two expert gastrointestinal pathologists (L.X. and N.L.). Discordant cases were reviewed jointly until a consensus was reached. The following parameters were evaluated for invasive lesions: depth of invasion (stage), degree of differentiation (grade), lymph-vascular infiltration, submucosal invasive depth (distance from the bottom of muscularis mucosa to the base of cancer nests) [4].

### Diagnostic criteria

**Figure 1:**
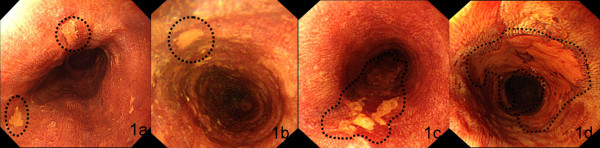
**Endoscopic images for the USL grade to diagnose the lesions.** The normal staining: Normal mucosa is negative after staining, and some very weak staining suggests esophagitis **(a)**. The USL is weak and the margin of the USL is indistinct, defined as the grade III, which is usually the mild dysplasia **(b)**. The USL is weak while the margin clear, defined as grade II, which is usually moderate dysplasia **(c)**. The USL is distinct with the margin clear, defined as grade I, with or without protrude or depressed lesions, which is usually the severe dysplasia (Tis) or T1 **(d)**.

Endoscopic diagnosis was based on the criteria established in esophageal cancer high-risk regions (Figure 1) [5]. After staining with Lugol’s solution, the mucosa with USL turned brown, defined as negative. Weak USL with indistinct margins, defined as grade III, was usually LGIN. The weak USL with clear margin defined as grade II, was usually MGIN. HGIN or T1 lesions comprise distinct USL with clear margin, defined as grade I, with or without protruded or depressed lesions.

EUS diagnosis was based on the layers of esophagus, to detect the invasive depth of the lesion into mucosa or submucosa, for possible resection. The EUS with the Sonoprobe System was used before ER to evaluate the resected specimens histopathologically. We interpreted the depth of tumor invasion based on ultrasonography using 20-MHz probe.

The macroscopic classification was based on the Paris endoscopic classification [2, 3], classified as 0-IIa, 0-IIb, 0-IIc, to distinguish the invasive from non-invasive lesions. The Paris classification dictated the choice of the ER.

Pathological diagnosis was based on a three-tier classification of squamous IN: LGIN, confined to the lowest third of the epithelium; MGIN, the lower two-thirds and HGIN, IN including the whole epithelium.

The pre-ER diagnosis was based on the worst possible diagnostic outcome among the endoscopic examination, the Paris classification and the biopsy histopathological diagnosis. Such as, the lesion was 0-II according to the Paris classification which was the indication for the ER. If the diagnosis based on the biopsy was HGIN, while endoscopic diagnosis after the lugo’s staining as the MGIN, the pre-ER diagnosis was HGIN with the EUS no invasion to submucosa. If the diagnosis based on the biopsy was MGIN, while endoscopic diagnosis after the lugo’s staining as the HGIN/ESCC, the pre-ER diagnosis was HGIN/ESCC with the EUS no invasion to submucosa.

The gold standard for the lesion was based on a combination of biopsy and ER, the worse one between these two diagnose was the gold standard.

The under-diagnosed rate was determined, based on the pre-ER systems or the ER diagnosis compared with the gold standard. The accuracy rate was determined, based on the pre-ER systems matching the gold standard. The over-diagnosed rate was determined, based on the extent to which pre-ER systems or the ER diagnoses were over-diagnosed compared with the gold standard.

### Statistical analysis

The under-diagnosis, accuracy, and over-diagnosis rate were calculated based on the number of the pre-ER as numerator and the gold standard as denominator. The under-diagnosis means the numerator was lesser than the denominator, while the accuracy means the same diagnosis, and the over-diagnosis means the numerator worse than the denominator.

The Receiver-Operating-Characteristic (ROC) curve was used to evaluate the accuracy of the pre-ER system and the ER diagnosis. The Area under each ROC (AUC) and its 95% confidence were calculated. We used the Z-test to examine the differences between the AUCs based on the pre-ER diagnosis and ER diagnosis. All statistics were performed using SPSS 17.0 for Windows**.**

## Results

**Figure 2:**
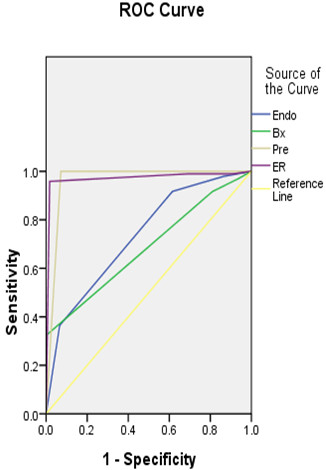
**The ROC of the Pre-ER system compared with the gold standard (Endo: the endoscopy diagnosis; Bx: the diagnosis based on the biopsy; Pre: the diagnosis based on the pre-endoscopic system).**

**Figure 3:**
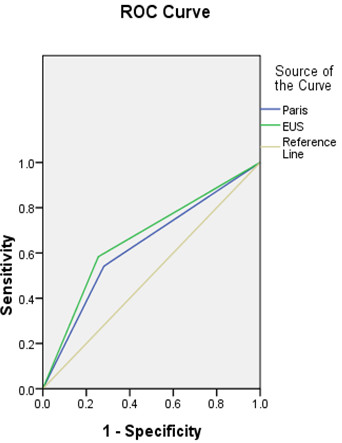
**The ROC of the Paris classification and the EUS compared with the gold standard in the staging (Paris: the Paris classification, EUS: endo-ultrasonography).**

The data included 217 lesions of 203 cases, 150 males and 53 females, aged between 31 and 80 years, with the median age 60 (Table 1). The esophageal cancer was mainly localized to the middle esophagus (Table 1). The pre-ER system included diagnosis based on biopsy, endoscopy, the Paris-classification and the EUS. We first analyzed the diagnostic accuracy of these modalities, individually. The under-diagnosed rate, the accuracy rate and the over-diagnosed rate based on biopsy was 38%, 62% and 0%, compared with the gold standard, respectively. The rated of diagnosis based on endoscopy were 49%, 46%, and 5%, compared with the gold standard, respectively. The diagnostic rates following the Paris-classification were 42%, 49%, 9%. Based on the EUS, they were 26%, 57% and 17%, and based on the pre-ER system 3%, 91% and 6%, respectively. The under-diagnosed rate, the accuracy rate and the over-diagnosed rate based on the ER-specimen were 7%, 93% and 0%, compared with the gold standard, respectively (Table 2).We calculated the AUC to quantify the consistency of these diagnoses. The AUCs of the endoscopic diagnosis, the Paris classification, the EUS and the biopsy were 0.738, 0.634, 0.683, and 0.696, respectively (P < 0.05). The AUC of the pre-ER system combined with the endoscopic diagnosis, Paris classification, EUS and biopsy was 0.963, while the AUC of the diagnosis based on the ER specimens was 0.971. No differences existed between these two AUCs (u = 0.0405, u <1.96, P >0.05) (Figure 2).The staging accuracy of the pre-system was evaluated using the AUCs of the EUS and Paris classification. The EUS was mainly used for evaluating the invasive depth, while the Paris classification was used for evaluating the invasive nature of the lesions, according to their endoscopic appearance. The AUCs of the EUS and the Paris classification were 0.683 and 0.634, respectively (Figure 3).

**Table 1:** **Clinicopathological characteristics of 217 lesions(203 patients)**

Characteristics	Number
Sex (male/female)	150/53
Age, median (range)	60 (31–80)
Location	
Upper Esophageal	21
Middle Esophageal	103
Lower Esophageal	94
The length of lesions (cm)	3-10
Histological diagnosis of ER specimens	217
LGIN	13
MGIN	28
HGIN	82
ESCC	94
Histological type of the final diagnosis	217
LGIN	0
MGIN	15
HGIN	106
ESCC	96

**Table 2:** **Summary of the comparison of the diagnoses based on the biopsy, the endoscopy, the EUS, Paris classification, ER specimens with the gold standard**

	Final Diagnosis						P value
	Pathological diagnosis				Invasive depth		
	LGIN	MGIN	HGIN	ESCC	Mucosa	Submucosa	
Biopsy diagnosis							<0.05
LGIN	0	4	5	3	NA	NA	
MGIN	0	11	7	5	NA	NA	
HGIN	0	0	94	57	NA	NA	
ESCC	0	0	0	31	NA	NA	
EUS							<0.05
Mucosa	NA	NA	NA	NA	143	11	
Submucosa	NA	NA	NA	NA	46	17	
Paris classification							<0.05
0-IIa	0	17	48	66	NA	NA	
0-IIb	0	14	89	150	NA	NA	
0-IIc	0	0	0	1	NA	NA	
Noninvasive	0	13	91	52	NA	NA	
Invasive	0	2	15	44	NA	NA	
Endoscopic diagnosis							<0.05
LGIN	0	7	11	2	NA	NA	
MGIN	0	3	27	6	NA	NA	
HGIN	0	4	61	53	NA	NA	
ESCC	0	1	7	35	NA	NA	
ER diagnosis							<0.05
LGIN	0	5	7	1	NA	NA	
MGIN	0	10	20	0	NA	NA	
HGIN	0	2	77	3	NA	NA	
ESCC	0	0	2	92	NA	NA	
Pre-ER diagnosis							<0.05
LGIN	0	0	0	0	NA	NA	
MGIN	0	9	6	0	NA	NA	
HGIN	0	4	93	0	NA	NA	
ESCC	0	2	7	96	NA	NA	

LGIN: low-grade intra-epithelia neoplasia; MGIN: middle-grade intra-epithelia neoplasia; HGIN: high-grade intra-epithelia neoplasia; ESCC: esophageal squmous cell carcinoma.

This study was approved by the Ethics Committee of Cancer Institute and Hospital, Chinese Academy of Medical Sciences with the Approval Number 13-069/745 with the written consent exempted.

## Discussion

Endoscopic resection (ER) is used to treat premalignant and malignant lesions of the digestive tract. ER for squamous superficial precursor of the esophagus was considered safe and efficient [10]. However, the pre-ER diagnosis was as important as the ER diagnosis for treatment choice, especially ablation therapies, such as the RFA and PDT.

It was demonstrated previously that the forceps biopsy was sometimes inadequate for accurate histological diagnosis [10, 11], while the ER was relatively precise (AUC = 0.971). Our data showed that the AUC based on biopsy was 0.695, that is, the diagnosis based on biopsy alone was not consistent with the gold standard. Our data showed the accuracy rate of the diagnosis based on biopsy was a mere 44% compared with ER-specimens. The discrepancy between the diagnoses between biopsy and the ER-specimens can be explained as follows. First, the biopsy tissue was a small piece of the mucosa. Although it may include the muscularis mucosa, it rarely contained the submucosa [12]. Therefore, the depth of the lesions cannot be accurately determined based on the biopsy. Polymorphism of the precursor was another important factor. Although the Lugol’s staining and the narrow band imaging (NBI) determined the extent of the lesions [13], the small tissue cannot depict the true characteristics of the lesions. Third, the management of the biopsy specimens affected the diagnosis of the lesion. If the biopsy specimens were not flat, the specimens shrunk with fixation, dehydration and embedding, resulting in poor orientation of the lesions, and thereby affecting the final diagnosis. Fourth, the subjective interpretation of the criteria among the inter-observers variation in precursor based on the biopsy have been a vexing issue for pathologists and clinicians, for a definitive diagnosis [14].

Our data showed that the AUC of the diagnosis based on the endoscopy and Paris classification were 0.735 and 0.728, respectively. The diagnostic consistency was better than the consistency based on the biopsy alone. The two approaches were based on endoscopic appearance using the chromoendoscopy, such as the Lugol’s staining. Lugol’s staining demonstrated the borderline and the stain grade, while the Paris classification distinguished the invasive from noninvasive lesion. It was reported that the “protruding” (type 0-I) and “excavated” (type 0-III) lesions were associated with higher risk of the submucosa invasion (70%) and lymph nodal metastases (30%) [3]. We used the Paris classification as the indication for the choice of ER. The invasion or non-invasion of the lesion should also combined with the magnifying endoscopy.

According to the follow-up data from the high-risk area, the rate of progression to esophageal SCC differs significantly among LGIN (5.3% at 3 years follow-up), MGIN (26.7%) and HGIN (65.2%) [15, 16]. Therefore, the lesions diagnosed as MGIN were also diagnosed by the ER. Endoscopy and the Paris classification enable the right choice of treatment. In our study, we adopted the worse outcome as the diagnostic strategy to design our treatment in the event of a discrepancy between endoscopy and biopsy. If the diagnosis based on endoscopy was HGIN and the pathological diagnosis based on the forceps biopsy was LGIN, the treatment should be discussed with the patients. The pre-ER diagnosis should combined these two diagnoses. The lesions diagnosed as HGIN should be examined using an ultrasonic probe to determine the invasive depth of the lesions. Nearly 45% (53/118) of the lesions diagnosed as HGIN were SCC based on the gold standard. The final treatment, such as the ER or esophagectomy, should follow the NCCN principles based on the pre-ER system diagnosis.

Using the EUS, the diagnostic accuracy for superficial esophageal carcinoma reached 93%, to differentiate mucosal from submucosal [17]. The 20 MHz probe showed the esophageal layers distinctly, with lesions invading the mucosa or submucosa scanned objectively [18]. Our data showed that the AUCs of the diagnosis based on the EUS and the Paris classification were 0.683 and 0.728 for staging, respectively, suggesting that the EUS and the Paris classification examined the invasive depth of the lesions objectively and enabled the choice of the treatment, combined with biopsy. However, 6.7%-35.2% of the early invasive esophageal cancers were associated with lymph node metastasis [19]. Using the EUS to evaluate the lesions, a false negative lymph node metastasis is a possibility, whereas T staging of superficial esophageal cancer was associated with 82.3% diagnostic accuracy [20]. Computed tomography and convex ultrasonography should be performed before ER to assess lymph node metastasis. During the EUS operational procedure, there was “upgrade” or “downgrade” rate. The reasons were that: (1) There was different appearance of the lesions and the different image in the EUS picture. When we used the probe to exam the depth of the lesion, there was bias because of this; (2) There was inflammation in the lesion, and the invasion depth affected; (3) There was minute invasion in the lesion and it was difficult to be tested in the EUS.

ER was considered more precise than the EUS with regard to the depth of invasion. Our data showed that the AUC of the pre-ER diagnosis was 0.968. No significant difference was observed with the AUC of the diagnosis based on ER, 0.971. Data showed that endoscopic diagnosis using the Lugol’s staining, Paris classification, EUS and biopsy were all needed before the ER, to arrive at an appropriate choice of treatments in early esophageal cancer. Similar approach is required for precancerous lesions using ER, ablation or esophagectomy. The pre-ER system was more important in the choice of the treatment in the absence of ER specimens, including the RFA and PDT indicated for the superficial esophageal precursor.

We tried to improve the diagnosis criteria by adding the grading of the endoscopic appearance of the lesions after Lugol’s iodine staining (based on the intensity of the lack of staining and the margin characteristics of the lesion), and by adding the stage criteria based on the Paris Classification and EUS findings. We concluded that the pre-ER system can estimate lesion histology and stage as accurately as post-ER by combining data from the endoscopic appearance and biopsy diagnosis. And the most important were that the use of the EUS for the stage and the Paris classification for the including criteria. The weakness of this study was that the study on the endoscopic appearance of the lesion after the lugol’s staining was mainly from China, and there was also no other study on it. In this study, we want to share the experience in the lugol’s for the diagnosis in the esophageal precursor.

In our study, we improve the diagnosis on these criteria by adding (1) a grading of the endoscopic appearance of the lesions after Lugol’s iodine staining (based on the intensity of the lack of staining and the margin characteristics of the lesion), and (2) the choice criteria for the ER based on the Paris Classification and EUS findings. The results showed that it can estimate lesion histology and stage as accurately pre-ER as post-ER by combining data from endoscopic grading of the Lugol’s unstained lesions, macroscopic appearance of the lesions by the Paris Classification, EUS findings, and biopsy diagnoses. In summary, the lesions which were diagnosed as early cancer or precancerous lesions a endoscopically should be re-diagnosed, based on a combination of the endoscopic diagnosis, the Paris classification, the EUS and the biopsy. The diagnostic accuracy of pre-ER system was comparable to ER, which was important for the ablation.

## Conclusion

In this study, we compared the diagnostic concordance of this pre-ER system and ER histological diagnosis with the gold standard in superficial esophageal precursor and to evaluate the accuracies. The result was inspiring that the system had the same accuracy with the ER specimen, and could make up the insufficient of the ER in the diagnosis for the lesions and help to select treatment for the superficial esophageal precursor.
